# Editorial: Molecular characterization of humic substances and regulatory processes activated in plants, volume II

**DOI:** 10.3389/fpls.2024.1413829

**Published:** 2024-04-23

**Authors:** Serenella Nardi, Michela Schiavon, Adele Muscolo, Diego Pizzeghello, Andrea Ertani, Luciano Pasqualoto Canellas, Jose M. Garcia-Mina

**Affiliations:** ^1^ Dipartimento di Agronomia, Animali, Alimenti, Risorse Naturali e Ambiente (DAFNAE), Università degli Studi di Padova, Legnaro, Padova, Italy; ^2^ Dipartimento di Scienze Agrarie, Forestali e Alimentari (DISAFA), University of Torino, Grugliasco, Italy; ^3^ Department of AGRARIA, “Mediterranea” University, Reggio Calabria, Italy; ^4^ Núcleo de Desenvolvimento de Insumos Biológicos para Agricultura (NUDIBA), Universidade Estadual do Norte Fluminense Darcy Ribeiro (UENF), Campos dos Goytacazes, Rio de Janeiro, Brazil; ^5^ Department of Environmental Biology, Sciences School, University of Navarra, Pamplona, Spain

**Keywords:** humic substances, molecular structure, biostimulants, plant growth, iron nutrition

The excessive use of synthetic fertilizers in agriculture over recent decades has had detrimental effects on soil properties, particularly in terms of reducing organic matter content and diminishing the soil microbial activity and biodiversity. Overuse of fertilizers has also increased the risk of nutrient losses through leaching into the soil, groundwater drainage, or surface runoff, thereby posing a threat to the environment. In addition, high fertilizer input can cause an imbalance in nutrient availability in the soil, leading to low nutrient use efficiency (NUE) and metabolic alterations, as plants may not be able to adequately absorb ad manage all the supplied nutrients. These adverse effects can be further intensified by ongoing climate changes, creating a scenario that requires the adoption of sustainable agricultural practices aimed at reducing the input of nutrients through fertilizers by optimizing the use of nutrients by plants and reducing their dependence on chemical fertilizers. In this context, biostimulants have attained growing interest in the last few decades, and their market has raised remarkably on a global scale. Among biostimulants, humic substances (HS) have a longer history. Their capacity to stimulate plant growth and nutrition through diverse mechanisms, both acting in the soil and within the plant, has been thoroughly ascertained. However, part of the mechanisms explaining their action and their effects still needs to be completely elucidated in small- scale and in field trials. Recent studies focusing on both these topics are included in the second volume of this Research Topic.

The study conducted by Lamar et al., in particular, was aimed to elucidate the functional groups within the structure of HS responsible for their biological activity. Specifically, the authors investigated the role of COOH and ArOH groups in determining HS biostimulant activity. Specifically, they used a humic acid (HA), purified from oxidized sub-bituminous coal to prepare HAs with COOH groups methylated (AHA), ArOH groups acetylated (OHA), and with both COOH and ArOH groups methylated (FHA). NHA was the original, non-modified HA. The four HAs were subjected to elemental, 13C-Nucleic Magnetic Resonance (NMR), Fourier Transform Infrared Spectroscopy (FTIR), and Electron Spin Resonance (ESR) analyses and their antioxidant properties were assessed using the trolox equivalents antioxidant capacity assay (TEAC). Results from 13C-NMR and FTIR analysis revealed significant alkylation/acetylation. A plant bioassay employing maize (*Zea mays* L.) seedlings, grown in either nutrient or non-nutrient stress regimes, was performed to evaluate the effects of the chemical treatments on humic fraction structures. Interestingly, the most pronounced effects on seedling growth were observed in corn seedlings subjected to nutritional stress conditions compared to those grown in nutrient-rich solutions. With respect to the HAs fractions endowed with alkylated/acetylated groups, the authors found that they exhibited growth effects similar to those observed with NHA, suggesting that neither COOH nor ArOH groups play a significant role in the biological activity of humic acid in this study.

In natural environment, the role of HS in promoting plant growth has been largely described, but the missing information is about the initial event triggered by the interaction between plant and HS. Possibly, this interaction may lead to significant modifications in the molecular conformation of humic self-assembled aggregates, which can consequently influence root responses. Aranaz et al. in their study assayed the capacity of a natural humic acid (HA) and a transformed humic acid obtained from the treatment of HA with fungal laccase (HA enz), to affect plant growth and copper (Cu) nutrition. The laccase treatment increased hydrophobicity, compactness, stability, and rigidity of HA enz without altering its molecular size. HA was found to stimulate shoot and root growth in cucumber and Arabidopsis, while HA enz did not elicit the same response. Moreover, there were no changes observed in Cu complexation features, and no molecular disaggregation occurred when HA and HA enz interacted with plant roots. Instead, both HA and HA enz underwent structural alterations, characterized by enhanced compactness and rigidity, perhaps facilitated by root exudates inducing intermolecular crosslinking. In conclusion, the results of this study indicated that the weakly bond stabilized aggregated conformation (supramolecular-like) of humic acid significantly contributed to its capacity to promote root and shoot growth. Furthermore, this study highlighted the presence of two main types of HS in the rhizosphere corresponding to those that do not interact with plant roots and form aggregated molecular assemblies, and those generated after interacting with plant root exudates and forming stable macromolecules.

Going into more details on the mode of action of HS at the root level, Garnica et al. investigated the action of humic acids on specific mechanisms activated in roots of plants experiencing Fe deficiency. The authors targeted graminaceous plants, as previous investigations in this area have been limited, with most of them focusing on dicotyledonous plants, where the ability of humic acids to enhance Fe solubility and bioavailability has been established. More specifically, in this study the authors evaluated the potential of a peat-extracted humic acid (HA) to enhance Fe nutrition in both Fe-deficient and Fe-sufficient wheat plants. Humic acid was shown to ameliorate the physiological status of Fe-deficient plants, correlating with the increase in the Fe-active pool in leaves, possibly facilitated by the mobilization of Fe complexes by HA through interaction with Phytosiderophores released into the nutrient solution. Furthermore, the Fe root to shoot translocation was likely facilitated by the presence of the phytohormone trans-Zeatin Riboside (tZR), the concentration of which in leaves was particularly elevated in Fe-deficient plants treated with HA.

While the effect of humic substances on crops has been studied for long time in small scale experiments and still receives a lot of attention, the study of the effects of humic substances on plants grown in open fields remains largely unexplored. Even less known are the biochemical changes that occur in plants treated with HS. In the study by Olk et al., the authors described the presence of 11 phenol and five carbohydrate monomers in the whole plant stover, over four growing seasons, and roots, over two growing seasons, of maize. These determinations were made at physiological maturity in two rainfed fields in Iowa (USA) after HS application. During the drier season of root sampling in 2013, the authors noted a respective increase of 12% and 19% in total phenols in plants following the two treatments with HS. However, no discernible effect was observed for the five carbohydrate monomers in the upland roots. In 2014, a year marked by high rainfall, a contrasting pattern was observed: root phenols remained unaffected by humic treatment, whereas root carbohydrates exhibited a notable increase of 11% or 20% after the two HS treatments, compared to the control. Phenols particularly responsive to either HS application or drier conditions included p-coumaric acid and syringaldehyde, both of which are significantly involved in late-season lignification process of maize. Concluding, in this study the authors found that the treatment of HS positively influenced the lignification process, which is a natural response to drought conditions; in non-drought conditions, the application of HS prompted an increase in root carbohydrate production, suggesting an intrinsic response induced by HS in plants under no stress conditions. These findings underscore the complexity of the various responses elicited by treatment with HS in the plant-soil-environment.

In conclusion, these manuscripts effectively emphasize the significant role of HS in enhancing plant growth and nutrition across various growth conditions. Many effects induced by HS depend on their molecular structure, as evidenced by induced molecular structural modifications, and their interactions with roots. Root exudates have the potential to alter the molecular conformation and properties of HS, while HS, in turn, can influence the release of specific exudates, such as phytosiderophores in Fe-stressed plants. Understanding the mode of action of HS in long-term field trials remains a compelling challenge for scientists, necessitating further investigation.

## Author contributions

SN: Writing – original draft, Writing – review & editing. MS: Writing – original draft, Writing – review & editing. AM: Writing – review & editing. DP: Writing – original draft, Writing – review & editing. AE: Writing – review & editing. LC: Writing – review & editing. JG: Writing – original draft, Writing – review & editing.

